# Assessing REM Sleep Behaviour Disorder: From Machine Learning Classification to the Definition of a Continuous Dissociation Index

**DOI:** 10.3390/ijerph19010248

**Published:** 2021-12-27

**Authors:** Irene Rechichi, Antonella Iadarola, Maurizio Zibetti, Alessandro Cicolin, Gabriella Olmo

**Affiliations:** 1 Department of Control and Computer Engineering, Politecnico di Torino, 10129 Turin, Italy; gabriella.olmo@polito.it; 2 Sleep Disorders Center, Department of Neuroscience, University of Turin, 10126 Turin, Italy; antonella.iadarola@unito.it; 3 Department of Neuroscience, University of Turin, 10126 Turin, Italy; maurizio.zibetti@unito.it

**Keywords:** machine learning, REM Sleep Behaviour Disorder, electromyography, Parkinson’s disease, RSWA, RBD detection

## Abstract

**Objectives:** Rapid Eye Movement Sleep Behaviour Disorder (RBD) is regarded as a prodrome of neurodegeneration, with a high conversion rate to α–synucleinopathies such as Parkinson’s Disease (PD). The clinical diagnosis of RBD co–exists with evidence of REM Sleep Without Atonia (RSWA), a parasomnia that features loss of physiological muscular atonia during REM sleep. The objectives of this study are to implement an automatic detection of RSWA from polysomnographic traces, and to propose a continuous index (the Dissociation Index) to assess the level of dissociation between REM sleep stage and atonia. This is performed using Euclidean distance in proper vector spaces. Each subject is assigned a dissociation degree based on their distance from a reference, encompassing healthy subjects and clinically diagnosed RBD patients at the two extremes. **Methods:** Machine Learning models were employed to perform automatic identification of patients with RSWA through clinical polysomnographic scores, together with variables derived from electromyography. Proper distance metrics are proposed and tested to achieve a dissociation measure. **Results:** The method proved efficient in classifying RSWA vs. not-RSWA subjects, achieving an overall accuracy, sensitivity and precision of 87%, 93% and 87.5%, respectively. On its part, the Dissociation Index proved to be promising in measuring the impairment level of patients. **Conclusions:** The proposed method moves a step forward in the direction of automatically identifying REM sleep disorders and evaluating the impairment degree. We believe that this index may be correlated with the patients’ neurodegeneration process; this assumption will undergo a robust clinical validation process involving healthy, RSWA, RBD and PD subjects.

## 1. Introduction

Neurodegenerative disorders represent a dramatic human and social burden. The increasing incidence and prevalence of Parkinson’s disease (PD) and Alzheimer’s dementia, just to mention the most common conditions, entail enormous direct and indirect costs for both families and healthcare systems [1,2]. Even though a causal therapy for these diseases does not currently exist, emerging evidence points out that life style modification can dramatically slow down the disease progression [3,4]. Moreover, new neuro-protective drugs are under study [5], which will be likely effective only if administered in very early, pre-clinical stages of the pathology [6]. This points out the need for early markers of neurodegeneration. This is also crucial for autonomic nervous system impairment, leading to orthostatic hypotension in PD patients [7].

In this context, sleep disorders represent a remarkable source of information. REM Sleep Behaviour Disorder (RBD) [8] is a parasomnia characterised by lack of physiological muscle atonia during REM sleep, which results in a dream-enacting and ofttimes violent behaviour. RBD is considered a prodrome of neurodegenerative disorders such as PD, Dementia with Lewy Bodies and multiple system atrophy [9,10]. In fact, the estimated risk of overt neurodegeneration is around 97% at a 14-year follow-up, while the conversion rate to PD is about 90% [11]; many clinicians consider RBD itself as an initial-stage neurodegeneration. It is worth noticing that the prevalence of RBD is 0.5 to 1.25% in the general population and approximately 2% in older adults; however, the vast majority of cases go unrecognised [12].

In accordance with the ICSD-3 criteria [13], the diagnosis of RBD requires the presence of both dream enacting, evaluated through sleep quality questionnaires, interviews and reported abnormal behaviour (e.g., by a bed partner), and REM Sleep Without Atonia (RSWA), i.e., lack of regular muscle atonia during REM sleep. This latter is normally detected in polysomnography (PSG) as an increased and persistent electromyographic (EMG) activity of the mylohyoid muscle. With respect to RBD, the detection of RSWA has the advantage of not relying on anamnestic information. Moreover, some studies claim that RSWA itself can be considered an early bio-marker of RBD phenoconversion [14], thus further determining the importance of its accurate assessment in view of identifying neurodegeneration markers.

PSG, which represents the gold standard in sleep studies, is forcefully involved in the diagnostic process of RSWA and RBD, which consequently turns out to be very time- and resource consuming, besides being prone to inter- and intra-operator variability. The first objective of the present study is to make the diagnosis of RSWA easier and less time-consuming, in order to allow early detection of RSWA.

The state-of-the-art approaches for RSWA scoring rely on the amplitude characteristics of the EMG signal during REM sleep (duration of muscle tone and twitches, in the time domain). Visual scoring is provided by the Montréal [15,16] and SINBAR methods [17], which analyse the amplitude and burst duration of the EMG signal during REM sleep in 2(3)-second epochs, respectively. Automatic scoring of RSWA has been proposed as well, through different indices [18,19,20,21]. They account for the amount of EMG activity during REM sleep, and are evaluated on 1-s or 3-s mini-epochs, depending on the implemented method. An exhaustive comparison of these metrics is provided in [22]. According to the cited study, the REM Sleep Atonia Index, introduced in [18], proves to be the most effective for the detection of RSWA.

A step forward in automatic sleep analysis has been achieved through Machine Learning (ML) techniques. Automatic classification of sleep disorders has been undertaken in [23], where RBD identification has been carried out, using polysomnographic, EMG and electro-oculogram (EOG) data [24]. However, the proposed classification algorithm is complex, as it involves several different signals and a high number of features.

In this paper, we provide a method for blind, automatic identification of RSWA in polysomnographic records, based on the spectral analysis of EMG record during REM sleep. We implement ML methods to classify a subject as affected by RSWA or not. Then, we introduce a continuous measure, named the Dissociation Index (DI), that quantifies the degree of impairment of the subject using Euclidean distance measures in proper vector spaces. Indeed, RSWA entails a state dissociation between mind and body; in fact, while the EEG reports the subject being in REM stage, the motor neurons are active and excitable [25]. Describing the degree of dissociation is of great interest in the clinical practice and can ease longitudinal evaluations. Even though it requires deep validation, the DI may represent a pilot to a finer evaluation and monitoring of RSWA and its progression towards neurodegeneration.

## 2. Materials and Methods

### 2.1. Subjects and Data

In this paper, we employ an internationally approved dataset, publicly available on PhysioNet [26], a repository of freely-available medical research data managed by the MIT Laboratory for Computational Physiology (https://physionet.org, accessed on 15 March 2021). The employed dataset is the Cyclic Alternating Pattern (CAP) Sleep Database [27], which comprises polysomnographic recordings of 22 RBD subjects (19 males, aged 70 ± 6 years) and 16 healthy subjects (HS, 9 males, aged 32 ± 5 years). Fourteen subjects in the CAP Sleep Database present with idiopathic RBD (iRBD); the remainder suffer from secondary RBD and are further divided in RBD with PD (PD-RBD, 6 subjects), Dementia with Lewy Bodies (LBD, 1 subject) and Multiple System Atrophy (1 subject). One subject in the HS group was excluded from further processing due to lack of EMG recording and other three HS were discarded due to the presence of ECG artifact, completely overlapping the submental EMG signal. A check on the duration of REM sleep was performed for all subjects in both the HS and RBD groups; all recordings presented with more than 5 min of REM episodes.

An additional private dataset (TURIN Database) was employed in order to test the robustness of the implemented algorithms. It encompasses full-PSG recordings of 18 clinically diagnosed or suspected RSWA subjects (11 males, aged 40 ± 20 years), for which the diagnosis was confirmed after PSG. One subject was undergoing treatment for PD at the time of the study. Inclusion criteria were: suspected narcolepsy or REM dissociation, suspected NREM parasomnias, secondary RBD due to Obstructive Sleep Apnea (OSAS) and increased EMG activity during sleep. Exclusion criteria included dementia or other psychiatric conditions preventing the correct execution of the PSG exam. Data were collected at the Center for Sleep Disorder of the Hospital “Città della Salute e della Scienza di Torino”, Turin, Italy. The procedure has been conducted in accordance with the Declaration of Helsinki and approved by the Ethics Committee of A.O.U. Città della Salute e della Scienza di Torino (Approval No. 00384/2020). The participants received detailed information on the study purposes and execution, and informed consent for observational study was obtained. PSG records were manually scored by sleep experts, who also identified events during sleep (e.g., arousal-related increased muscle tone during REM Sleep), which were considered as artefacts and excluded from the subsequent analysis.

### 2.2. The Implemented Algorithms

In this section, the implemented classification algorithms and the Dissociation Index are discussed in detail. The data processing and the algorithm software implementation were performed in Matlab 2020b and Python languages.

#### 2.2.1. Feature Extraction

First of all, a set of proper features to be input to the classification algorithms had to be identified. In this study, commonly employed polysomnographic variables [28] are taken into consideration and computationally extracted from the manually annotated hypnogram. They include the Sleep Onset Latency (SOL), Wake After Sleep Onset (WASO), Total Sleep Time (TST), Time in Bed (TIB), Sleep Efficiency (SE), Arousal Index (ARI), Minutes of REM Sleep (MREM); detailed definitions are provided in Table 1. Moreover, following [29], other polysomnographic features have been included in our analysis, namely: average length and proportion of segments classified as belonging to the same sleep stage, Sleep Transition Index (STI), REM and non REM (NREM) Fragmentation Indices (RFI and NFI). These latter measure sleep fragmentation patterns, typical of poor sleepers [29]. Again, descriptive definitions are reported in Table 1.

Other significant features are worked out from EMG data. The *REM Sleep Atonia Index* (RAI) [18] assesses the level of atonia during REM sleep. It is computed on the submental EMG signal on 1-s mini-epochs, and accounts for the percentage of mini-epochs with amplitude lower than 1 μV. The index ranges in [0, 1], with 1 denoting physiological sleep conditions. Recent studies [30] claim that the RAI may serve as a first-line method for revealing RSWA.

Finally, the set of considered features encompasses novel information from the spectral analysis of EMG during REM sleep. We selected 1-second mini-epochs, to match the RAI computation, and estimated the Power Spectral Density (PSD) using the Welch modified periodogram with Hamming window. Three features are then obtained: Mean Frequency of the power spectrum estimate, an averaged measure which represents the PSD centroid; Median Frequency (a.k.a. Spectral Edge Frequency at 50%, SEF50) representing the frequency below which 50% of the total power lies; Spectral Edge Frequency at 95% (SEF95), i.e., the frequency below which 95% of the total power lies. The complete list of features addressed in this paper, along with a brief description, is reported in displayed in Table 1.

All the features underwent z-score normalization and two-tailed *t*-test at the 5% significance level.

#### 2.2.2. Machine Learning Classification

**Figure 1:**
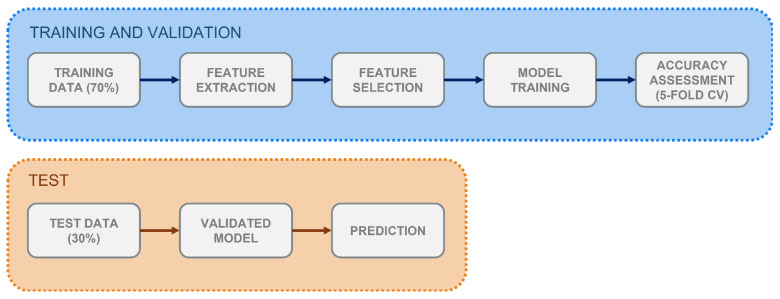
Workflow for the supervised classification process implemented in the study. Training and validation, test-set classification.

In this work, supervised learning methods were employed for the automatic identification of RSWA patients. These are algorithms which learn from the available known and properly labelled data (i.e., *training data*) and, through optimisations steps, build a model which can be later used to classify unknown data. A workflow of the process is shown in Figure 1. Given the aim of the study, the analysis was performed using manually (not automatically) scored PSG data to prevent any performance bias. Two ML models—K-Nearest Neighbour (K-NN) and Support Vector Machine (SVM)—were employed in a binary classification task between RSWA and non-RSWA subjects. These are distance-based algorithms described in the following.

- **K-NN** classifies each element by taking the majority vote on the class of its K closest items (i.e., *neighbours*) [31], where K is a parameter to be optimised.
- **SVM** aims at finding the hyperplane which effectively separates the elements in the dataset according to their class, by ensuring the maximum distance between the nearest items of each class [32].

Table 2 reports pivotal information regarding the ML models, as well as a summary of the main parameters and implemented optimisation steps. The applied normalization (always required for distance-based classifiers) and the feature selection method (necessary to lower the computational burden while ensuring good classification performance) are also displayed. Regarding feature selection, after a tuning phase, the 25th percentile was chosen as a variance threshold (var${}_{th}$ = 0.00087), keeping only those features whose variance was above var${}_{th}$; this yielded the best trade-off between the number of informative features and the number of predictors, and prevented over- and under-fitting. Finally, hyper-parameter optimisation has been performed. This is a crucial tool in ML, as it explores the possible algorithm configurations and selects the one which yields the best performance on the validation set. The list of optimised parameters is also included in Table 2.

To perform the analysis, the CAP dataset was partitioned into a training set (70%, 23 subjects, 8 HS) and a test set (30%, 11 subjects, 4 HS). Performance on the training data was assessed through *k*-fold Cross-Validation (CV), where *k* = 5. CV is a technique which allows to assess the generalization capability of the trained classifier—i.e., the ability to properly classify unseen data. It randomly splits the training set into *k* subsets, and, at each iteration, trains the model on k−1 subsets and validates it on the remaining one.

#### 2.2.3. The Dissociation Index

As discussed, RBD is considered precursor to neurodegeneration. Moreover, subjects that present more serious clinical manifestations of RSWA are more likely to develop RBD [14,33]. In fact, RSWA does not manifest itself at the same dissociation extent for all subjects. In light of these observations, we define the Dissociation Index (DI) as a distance-based continuous index correlated with the degree of sleep (and atonia) impairment. The DI functions as a similarity measure that compares a subject to a healthy model (*reference*, detailed after). The similarity between the subject and the reference is expressed by means of the Euclidean Distance (ED). A null distance denotes identity with the reference (i.e., perfect healthy conditions), whereas larger distance values denote increasing difference from the reference. To the best of our knowledge, this is the first study that proposes a distance-based model to assess the disease level.

**Figure 2:**
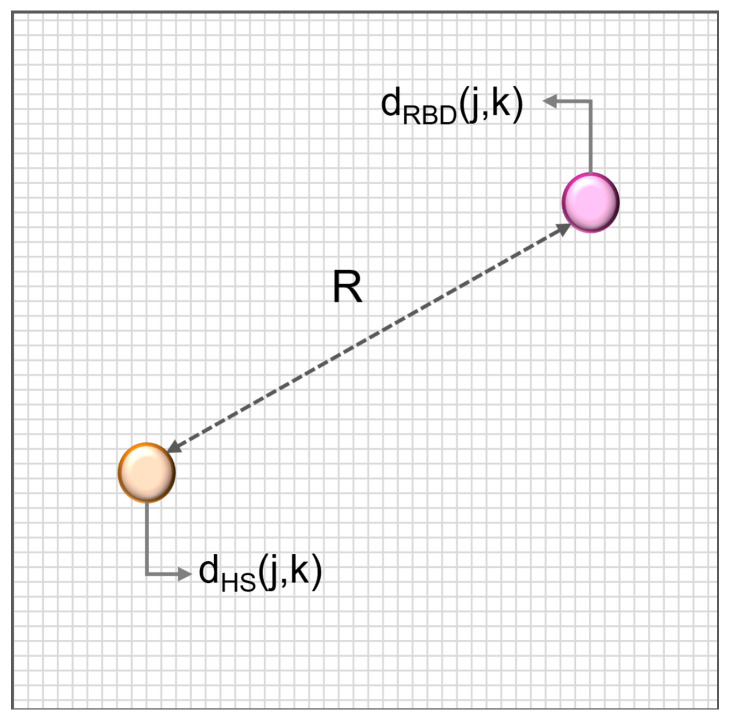
Neighbourhood R${}_{2}$ computation. It is the Euclidean Distance between two points in space, representative of the healthy subject (HS, orange) and RBD groups (purple) respectively.

In order to specify the DI metric, we define the concept of *neighbourhood*. This is not straightforward, given that there is no clinically validated disease model. In this work, two neighbourhood definitions are proposed, whose radius is evaluated as follows:

1. **Neighbourhood radius R${}_{1}$**: the ED between the HS with the best atonia score and the RBD subject with the worst atonia score. This defines a range between the best and the worst vectors in the dataset.
2. **Neighbourhood radius R${}_{2}$**: the ED between two points in space, representative of the HS and the RBD groups respectively (Figure 2). The two points are obtained as the ED of each group. This second definition is more exhaustive, as it does not depend on any single subject, but encompasses the characteristics of each whole group.

The values of the two resulting neighbourhood radii, worked out on the CAP Database, turn out to be R${}_{1}$ = 6.2 and R${}_{2}$ = 5.92.

Two reference vectors (expressed feature arrays) are proposed, consistent with the definition of the neighbourhoods:

1. **Reference S${}_{1}$**: the subject in the HS group that features the best atonia score;
2. **Reference S${}_{2}$**: the sample mean of all vectors in the HS group. This represents a more exhaustive measure which takes into account the variability within the HS cohort.

**Figure 3:**
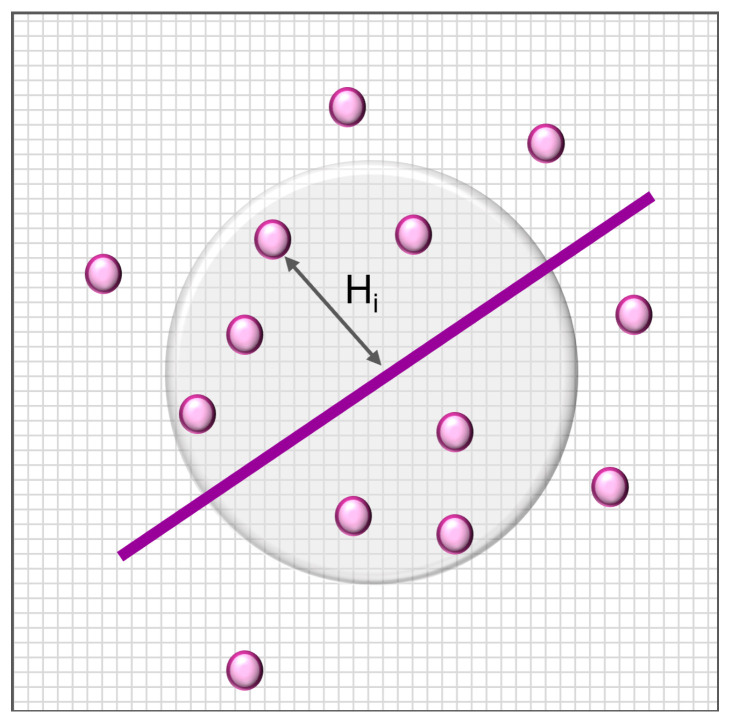
Proposed framework for assessing similarity. H${}_{i}$ is the euclidean distance between the i-*th* subject and the chosen reference.

Then, subjects lying in the R${}_{x}$ neighbourhood of the selected reference (x=1,2) are identified, and the distance H${}_{i}$ between the i-*th* subject and the reference vector is computed. Subjects lying outside the neighbourhood are automatically assigned the maximum value (i.e., the R${}_{x}$ radius), and are considered to be the most dissimilar from the healthy model (Figure 3).

Finally, distances $H_{i}$ are mapped to the Dissociation Index, in order to provide a normalized, independent and reproducible metric. The DI for the generic *i*-th subject lies in the range [0, 1], and is defined in Equation (1):

(1) $$DI_{i}=\frac{H_{i}-min}{max-min}$$

where, as previously stated, *H*${}_{i}$ represents the ED of the *i*-th subject, *min* is the minimum admissible distance value (i.e., 0), and *max* is the maximum distance value (i.e., the neighbourhood radius).

Subjects outside the neighbourhood are automatically assigned the value $DI_{i}=1$. Values of $DI_{i}$ close to 0 indicate strong similarity of the *i*–th subject to the reference (healthy model). Values close to 1 represent dissimilarity from the healthy model, hence increasing dissociation.

## 3. Results

In this section, we report the results achieved by the two classification algorithms, and an assessment of the Dissociation Index effectiveness in estimating the degree of impairment of subjects in our databases. Preliminary evaluations of some of the most relevant features considered in this work are also provided, to assess their suitability to drive the classification process.

### 3.1. Preliminary Feature Analysis: The RAI

**Figure 4:**
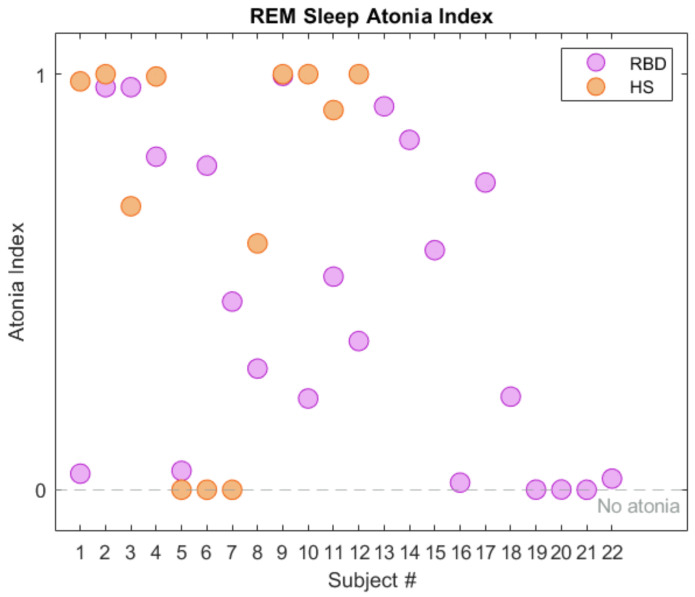
REM Sleep Atonia Index (RAI) in healthy subjects (HS, orange) and RBD (purple), in the CAP Sleep Database. The gray dashed line represents lack of REM atonia.

We recall that the REM atonia index ranges between 0 and 1. Values below 0.8 are strongly indicative of altered muscular atonia [18,30]; the [0.8, 0.9] range suggests slightly altered atonia, and values over 0.9 indicate a healthy condition. Working on the CAP dataset, the RAI (mean value ± standard deviation) turned out to be 0.93 ± 0.14 and 0.44 ± 0.37 in the HS and RBD groups respectively. This is in line with the results reported in [19]. As depicted in Figure 4, most HS exhibit RAI close to 1, denoting physiological sleep atonia. However, three subjects in the HS cohort (no. 5, 6, 7) exhibit null RAI; they have been excluded from the subsequent analysis. The RBD subjects present with higher RAI variability, leading to a quite large standard deviation. This strengthens the concept that dissociation of REM sleep occurs at diverse extents, and enforces the idea of a global index to describe the degree of impairment of each subject. A summary of the RAI statistical parameters is reported in Table 3.

**Figure 5:**
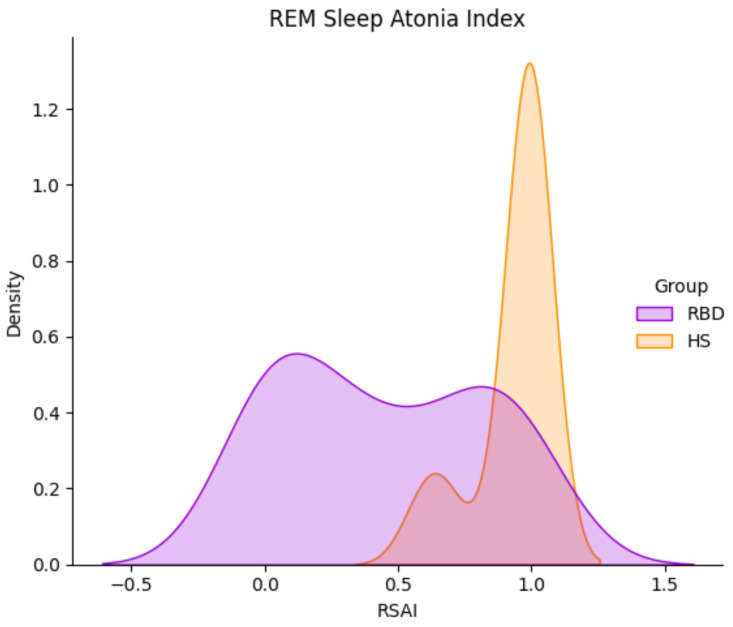
REM Sleep Atonia Index (RAI) Kernel Density Distribution in HS (orange) and RBD subjects (purple), in the CAP Sleep Database. RBD subjects present with a wider RAI distribution.

**Figure 6:**
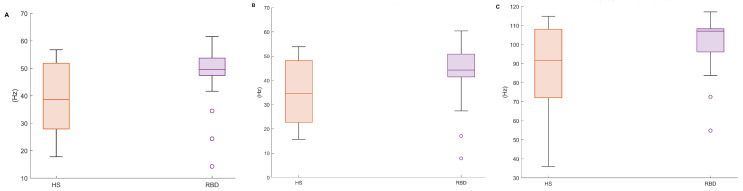
Boxplots of the proposed EMG spectral features, in both healthy (HS) and RBD subjects (RBD). (**A**) Mean Frequency, (**B**) Median Frequency, (**C**) SEF95. CAP database.

Figure 5 shows the RAI Kernel Density Estimation (KDE), computed for both HS and RBD sub-groups. Albeit displaying a narrow distribution, a few HS lie below the median value MED${}_{RAI}$ = 0.945. This gives rise to a wide interquartile Range (IQR, 75th–25th percentile = 0.7; see Figure 6), which, in turn, leads to high variance in the spectral features. Promoting this concept, only the spectral features of HS with RAI > MED${}_{RAI}$ were employed for classification.

### 3.2. Preliminary Feature Analysis: EMG Spectral Features

Table 4 and Figure 6 report the EMG spectral features addressed in this paper (MF, SEF50, SEF95—*cf.* Table 1) evaluated on the CAP Database. It can be noticed that the mean, median, and the 25th percentile considerably differ in the two populations. It is reasonable to assume that the higher variability in the HS accounts for the physiological diversity of the sample. Instead, RBD subjects display uniform characteristics within the group, as for these metrics.

**Figure 7:**
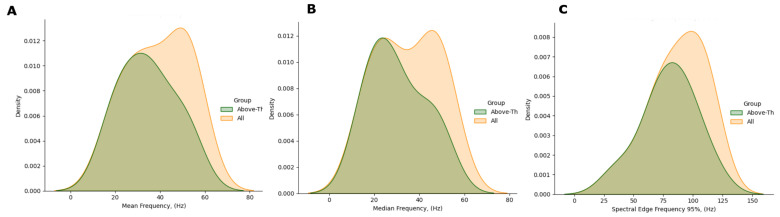
Comparison of the spectral features distribution plots, as regards all HS (yellow) and HS with RAI above threshold (green). (**A**) Mean Frequency, (**B**) Median Frequency, (**C**) SEF95.

**Figure 8:**
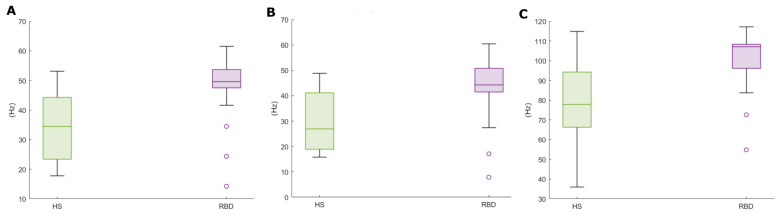
Spectral features boxplots, for above-threshold HS (green) and RBD subjects (purple). (**A**) Mean Frequency, (**B**) Median Frequency, (**C**) SEF95.

The plots in Figure 7 show the KDE distributions considering all subjects, and that evaluated on above—threshold subjects, i.e., those with $RAI>MED_{RAI}$—the latter are those subsequently considered in the analysis. As appreciable, the frequency distribution is rather even; besides, the max-min range is reduced in the Mean and Median Frequencies (Figure 8).

### 3.3. Classification: Cross-Validation Performance

Table 5 reports the 5-fold cross–validation results provided by the K–NN and SVM classification models, in terms of accuracy, sensitivity, specificity, precision (PPV) and false discovery rate (FDR). We compare our results with those in [24], which employs the same CAP dataset. The two models perform well on the training data. K–NN yields the highest overall accuracy (86.96%) and sensitivity to the RSWA class (93.33%), comparable with [24], which however employed a larger set of features. In fact, when performing RSWA detection by means of the atonia index only, the authors report an accuracy and sensitivity of 83% and 68%; these scores improve when the classification is performed through Random Forest (RF) employing a larger set of features (accuracy: 90%, sensitivity: 88%). On the other hand, our study exploits a minimal set of EMG spectral features alongside polysomnographic ones, yielding similar or higher sensitivity values. For completeness, a comparison of precision values (over 86% in our study) would be necessary; however, this information is not reported in [24].

### 3.4. Classification: Test Set Performance

Given the overall good performance of the K–NN classifier, this model was tested on the remaining set of data (i.e., 30% of the CAP dataset), comprising 11 subjects (4 HS). The results are shown in Table 6, which also displays the overall error measure (Mean Absolute Error, MAE). The model was optimised on the training data, and this justifies the slightly impaired performance. Nevertheless, the classifier proved very efficient in terms of overall classification accuracy (81.82%), sensitivity (85.71%) and PPV, 85.71%).

The model error was 13.63%, resulting in an average model accuracy (1 − MAE) = 86.37%. Furthermore, the model offers a good sensitivity/specificity trade-off (85.71% and 75%, respectively). The lower specificity is probably due to the smaller number of HS. Nevertheless, while sensitivity decreases in the test phase, specificity is stable, suggesting a good feature configuration against false positives. These results reflect the performance range for RBD detection presented in [24]. However, as previously stated, not only the cited study comprises a larger cohort (including RBD subjects from the CAP Database as well as a private set of data), but it also employs a higher number of features, both in time and frequency domains.

### 3.5. Classification Performance on the TURIN Database

**Figure 9:**
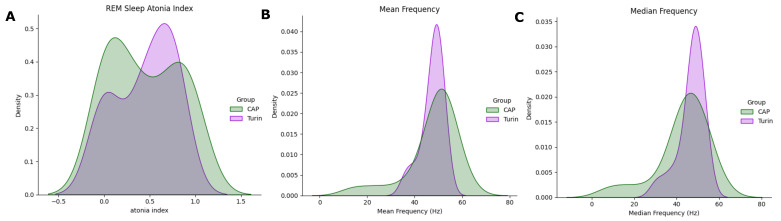
Distribution plots of three variables, computed in the CAP Sleep Database (green) and TURIN Database (purple). (**A**) RAI, (**B**) Mean Frequency, (**C**) Median Frequency.

The TURIN Database was employed in the testing stage of our algorithm. Feature extraction was carried out according to the methods in Section 2.2. The RAI values are coherent with those obtained on the CAP Sleep Dataset (TURIN: 0.45 ± 0.31, CAP: 0.45 ± 0.38). The atonia index distribution was compared in the two populations by means of the KDE (Figure 9). Likewise, the density distributions of the spectral features were compared (Figure 9b,c). As appreciable from the plots, data distributions in the muscular features reasonably overlap. This suggests that the proposed spectral features are appropriate for describing RSWA patterns also in the TURIN Database. Subsequently, the two previously trained ML models were tested on this additional database, to assess the ability of the proposed techniques to correctly detect RSWA subjects. Being a one-class assessment (i.e., no HS are available), the performance was evaluated in terms of MAE and average model accuracy (Table 7).

The results in Table 7 suggest that the models perform well on the new dataset. In more detail, SVM yielded a MAE of 5.55%, mis-classifying only one subject. On the other hand, K–NN was able to correctly classify all impaired subjects; however, it is reasonable to assume that the Model Accuracy would decrease when introducing a higher number of subjects.

### 3.6. Dissociation Index: Results

This section discusses the results obtained through the implementation of similarity measures, leading to the calculation of the DI.

#### 3.6.1. Neighbourhoods R_1_/R_2_, Reference Array S_1_

**Figure 10:**
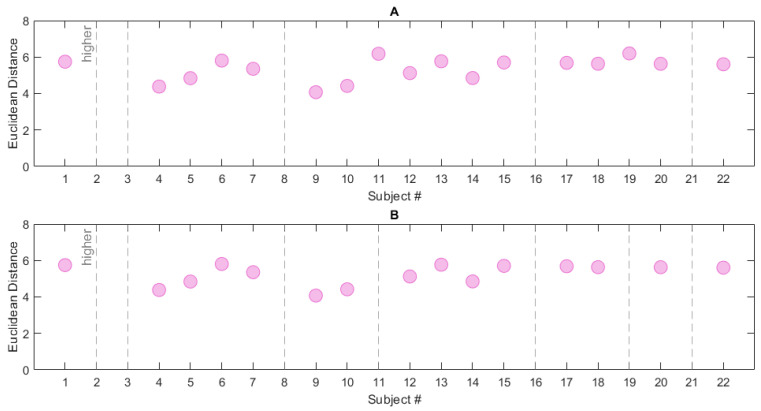
Euclidean Distance computed for the RBD subjects in the CAP Sleep Database. Two neighbourhood/reference combinations are reported: (**A**) R${}_{1}$/S${}_{1}$, (**B**) R${}_{2}$/S${}_{2}$. The grey dashed lines represent subjects located outside the neighbourhoods (i.e., subjects whose Euclidean Distance from the chosen reference is higher than the neighbourhood radius).

In order to define the DI, different neighbourhood/reference combinations were examined. The first considered reference was $S_{1}$ and it was tested with both neighbourhoods (i.e., radius R${}_{1}$ and R${}_{2}$). After the range search, 17 subjects of the CAP Sleep Database were found within neighbourhood R${}_{1}$ and 15 within R${}_{2}$; this may imply that this latter is more restrictive. Figure 10 shows the Euclidean distance from reference S${}_{1}$ of the RBD subjects in the CAP Sleep Database. 5 (7) subjects are located outside the radius R${}_{1}$ (R${}_{2}$) respectively. The ED trend is quite similar in the two cases, as appreciable from Table 8.

#### 3.6.2. Neighbourhood R${}_{2}$, Reference Array S${}_{2}$

In view of the results presented in Table 8, which show R${}_{2}$ to be the most restrictive, the neighbourhood was tested against the reference array S${}_{2}$—which, as previously stated, is a more exhaustive measure. Figure 10 reports the ED for each RBD subject; in this case, 6 subjects are found outside the neighbourhood radius. This is the most complete and selective measure, as it encompasses the characteristics of all subjects in the control dataset. The ED mean value and standard deviation are 5.02 ± 0.6 (maximum value: 5.81, number of excluded subjects: 6). Table 8 summarizes the information so far presented. To conclude, we may infer that the combination R${}_{2}$/S${}_{2}$ provides a robust measure for the evaluation of the dissociation, and a good trade-off between a restrictive neighbourhood and a complete set of data. Therefore, the values of ED obtained through this combination were used to compute the DI; the DI was assessed on the RBD subjects in the CAP Sleep Database.

**Figure 11:**
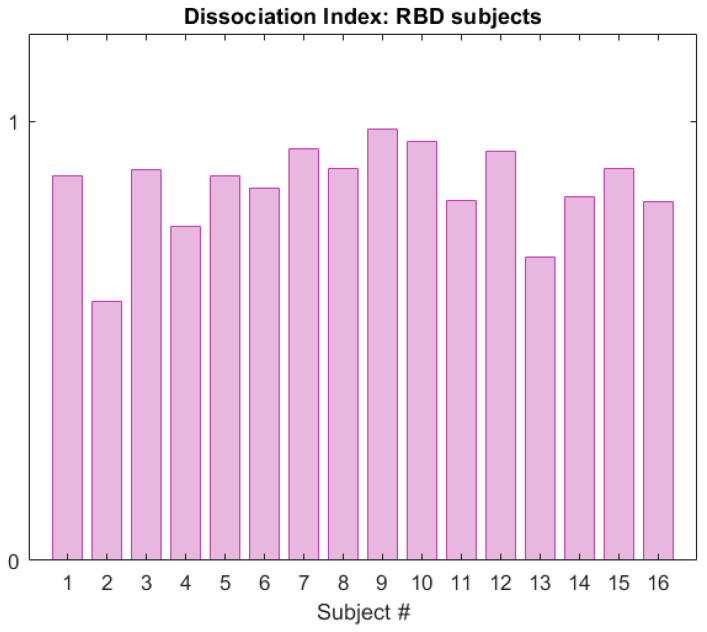
Dissociation Index computed on the RBD subjects (CAP Sleep Database), within neighbourhood R${}_{2}$ of reference S${}_{2}$.

**Figure 12:**
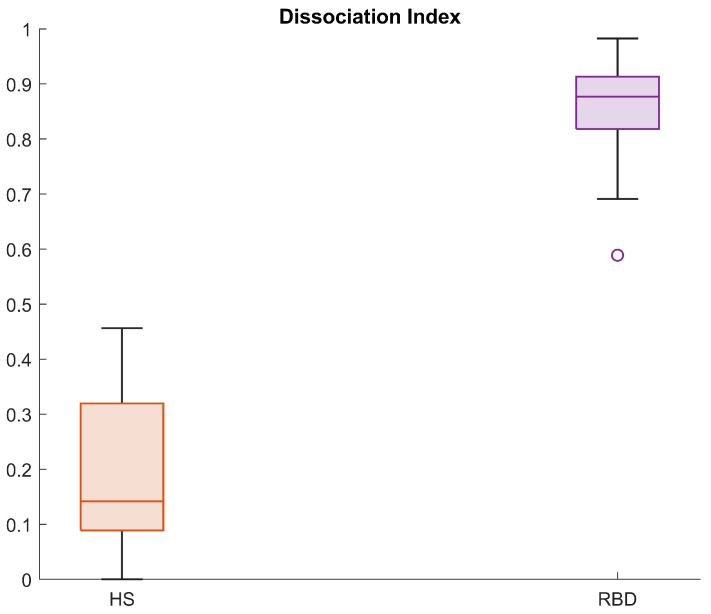
Boxplots for the Dissociation Index computed in the HS and RBD groups (CAP Sleep Database).

Figure 11 displays the DI calculated in the RBD group in the CAP cohort, for subjects belonging to the neighbourhood R${}_{2}$. The same framework was applied to the subjects in the healthy group, and Figure 12 shows a comparison of the DI data distribution for healthy versus RBD subjects.

As appreciable from Figure 12, the index distributions in the two groups considerably differ. Indeed, the 25th percentile in the HS group is 0.1 and the maximum value is below 0.5. On the contrary, the minimum value in the RBD group is 0.7 and the 75th percentile lies above 0.9, indicating great dissimilarity from the healthy model. These results suggest that the DI is a promising tool in the evaluation of the degree of dissociation, useful both for screening tests and longitudinal or follow-up procedures. Indeed, the physician may evaluate the DI at each follow-up, in order to see whether the index varies (for instance, increasing) or is stable over time. For these purposes, four areas—virtually corresponding to four impairment degrees—are preliminarily identified:

- **Low Tier** (LT): from 0 to 75th percentile of the DI in the healthy group (Q3${}_{HS}$),
- **Moderate Tier** (MT): from Q3${}_{HS}$ to the 75th percentile of the DI in the RBD group (Q3${}_{RBD}$),
- **High Tier** (HT): from Q3${}_{RBD}$ to 1,
- **Very High Tier** (VHT): above 1 (subjects who lay outside the considered neighbourhood).

**Figure 13:**
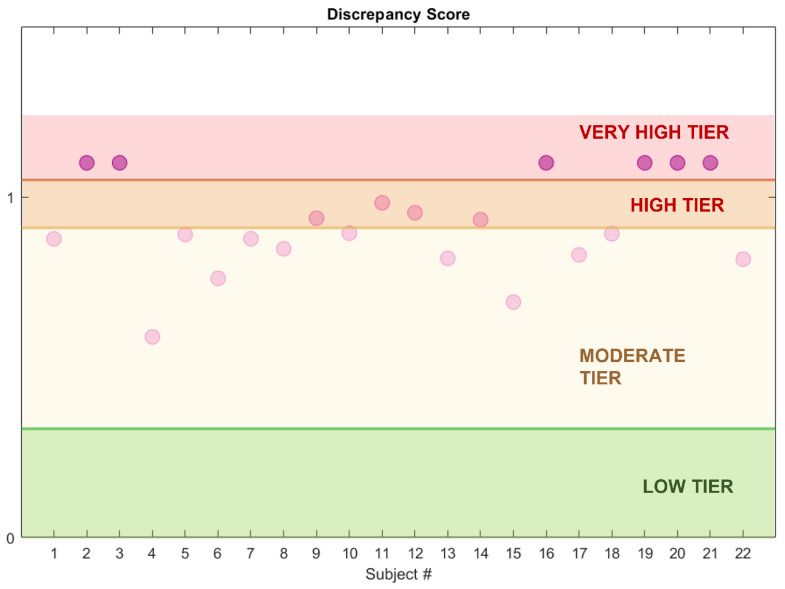
Dissociation Index and estimated impairment areas. The dots represent the subjects’ DI value (RBD cohort, CAP Sleep Database).

In principle, these ranges could help the physician monitor the disease progression over time, by locating each subject in the corresponding risk tier depending on the level of impairment (Figure 13). However, these results need further clinical validation.

## 4. Discussion and Conclusions

RBD is considered an early stage of neurodegeneration. Its diagnosis requires a full-PSG exam and clinical interviews, taking into account both quantitative and qualitative measures. Therefore, it is a long process, especially considering that it is usually performed after the symptoms’ onset, and leads only to pharmacological mainstays [34]. There is a clinical need to simplify the diagnostic process, and to effectively detect RSWA-like behaviours before their conversion to RBD in order to enact conservative treatments with the aim of forestalling disease progress [3]. Automatic measures have already been proposed to detect RSWA; however, these methods may easily be affected by artefacts, such as ECG or power-line noise. Moreover, amplitude-based methods estimate the amount of increased EMG activity during REM, but do not directly describe the degree of REM dissociation. From a clinical point of view, there is the need to assess the state dissociation in order to monitor its status at longitudinal follow-ups. This work proposed the automatic detection of RSWA manifestations, through clinical and polysomnographic parameters and three novel features based on the spectral characteristics of the submental EMG signal. The whole set of computationally extracted variables was input to ML supervised distance-based models (namely, K–NN and SVM) and proved effective in classifying RSWA vs. non–RSWA subjects. The K–NN classifier yielded the highest performance, with an overall accuracy of 87% and sensitivity to the target class (i.e., RSWA) of 93.33%. These metrics, along with a 75% specificity are encouraging, and also in line with previous work [24]. We have reason to believe that the models are robust and have good generalization capability, also given its performance on the test set and the additional test data. Finally, by further exploiting the concept of distance and similarity, this work introduced the DI, a distance-based index describing the degree of similarity to a healthy model. It can be adopted in the clinical practice to assess the extent of REM dissociation and, therefore, monitor the disease progress through a single parameter. From the available data, four impairment areas were proposed, from the LT to the VHT (i.e., where the REM dissociation is maximal). While being aware that the DI is a preliminary measure, we believe that it may have significant impact on screening and follow-up procedures, *de facto* working as a diagnosis support tool.

### 4.1. Possible Clinical Implications

The relationship between health costs associated with the diagnosis and treatment of sleep disorders and those of health complications directly related to the failure to treat them clearly favours the first approach. Some sleep components (e.g., muscle tone in REM phase) represents nowadays the most reliable early marker of PD, which affects about 1% of the population over 65, with an expected doubling in 2035 and an estimated healthcare cost of 2.2–2.9 billion Euros per year (data from the Italian Society of Neurology, 2021). Hence, it turns clear that the early detection of RBD (and other sleep alterations) and the possibility of starting early therapeutic options (from lifestyle modification to neuro-protective drugs under development), can contribute not only to reduce the epidemiological load of these disorders, but also to improve the quality and life expectancy of patients and the direct and indirect costs. Currently, sleep study methods (e.g., laboratory polysomnography and similar techniques implemented at home) are not suitable for a population screening, due to the intrinsic cost of the instruments and the time required to analyze the traces—which are commonly manually and visually scored by qualified technicians, as at present the qualitative level of automatic analysis methods does not meet the human gold standard. Hence, the identification of the polysomnographic alterations associated with the early detection of sleep disorders, and the design of automatic signal analysis tools can lead to the development of simplified, economically sustainable devices in terms of hardware and software, optimised for screening studies on large populations.

### 4.2. Limitations and Further Developments

This study is not without limitations. First, the DI requires clinical validation, performed in close cooperation with physicians, in order to test its accuracy, efficacy and reliability in the clinical and diagnostic practice. Preliminary assessment regarding this matter is currently underway. Moreover, as already mentioned, RSWA can not only manifest itself with different degrees of impairment, but also be related to pathologies other than RBD, and vary depending on age, medication and sleep habits. Therefore, there is a need to properly explore the diversified nature of RSWA, and future work will include in the study a larger cohort, encompassing subjects affected by RSWA, iRBD, PD-RBD, OSAS and age-matched controls. The experimental analysis and clinical validation of the index is currently ongoing.

## Figures and Tables

**Table 1:** Features employed in the study, along with their acronym and description. The extracted features are of three different kinds: polysomnographic (i.e., clinical), muscular (EMG) in the time domain, muscular (EMG) in the frequency domain. As for polysomnographic features referring to sleep stages, the following definition of sleep stage holds (according to the international guidelines): 1 (N1), 2 (N2), 3 (slow wave sleep or deep sleep, SWS) and REM sleep.

Type (or Channel)	Feature	Description
**Polysomnographic features**	Sleep Onset Latency (SOL)	The amount of time required to fall asleep (minutes)
	Wake After Sleep Onset (WASO)	The amount of time the subject is awake during the recording (minutes)
	Total Sleep Time (TST)	Total hours of sleep
	Time in Bed (TIB)	Lights-off to lights-on interval (hours)
	Sleep Efficiency (SE)	The ratio between TST and TIB (%)
	Arousal Index (ARI)	Frequency of occurrence of arousals
	Minutes of REM Sleep (MREM)	Total duration of REM Sleep (minutes)
	Proportion of N1 Sleep (PN1)	N1 sleep per TST (%)
	Proportion of N2 Sleep (PN2)	N2 sleep per TST (%)
	Proportion of SWS Sleep (PN3)	SWS sleep per TST (%)
	Proportion of REM Sleep (PNR)	Proportion of REM sleep per TST (%)
	NREM Fragmentation Index (NFI)	A measure of the number of transitions from NREM to any other NREM stage per hour of NREM sleep
	REM Fragmentation Index (RFI)	A measure of the number of transitions from REM to any other sleep stage per hour of REM
	Wake Proportion (WP)	Awake time during the night (%)
	Sleep Transition Index (STI)	A measure of the number of transitions from REM to NREM (and vice versa) per hours of sleep
	Average Length N1 (ALN1)	Average length of N1 segments (minutes)
	Average Length N2 (ALN2)	Average length of N2 segments (minutes)
	Average Length SWS (ALSWS)	Average length of SWS segments (minutes)
	Average Length REM (ALREM)	Average length of REM segments (minutes)
**EMG, time domain**	REM Sleep Atonia Index (RAI)	A measure of the amount of atonia during REM Sleep, evaluated on 1-s mini-epochs
**EMG, frequency domain**	Mean Frequency of REM mini-epochs (MF)	Mean Frequency of EMG signal during REM Sleep, 1-s mini-epochs (Hz)
	Mean Frequency of REM mini-epochs (SEF50)	Median Frequency of EMG signal during REM Sleep, 1-s mini-epochs (Hz)
	Spectral Edge Frequency at 95% of REM mini-epochs (SEF95)	Frequency below which 95% of the total spectral power is found on the EMG signal during REM Sleep, computed on 1-s mini-epochs (Hz)

**Table 2:** Summary of the two employed Machine Learning models: K-Nearest Neighbour (K-NN) and Support Vector Machine (SVM). For each classifier, the chosen feature selection and cross-validation methods, the searched hyper-parameters and the optimised model parameters are presented.

	K-NN	SVM
**Normalization**	z-score normalization	z-score normalization
**Feature Selection**	Variance Threshold (25th percentile)	Variance Threshold (25th percentile)
**Hyperparameters Search Range**	K: range [1:1:12]	Kernel Function: Linear, Quadratic,
	Distance Metric: City Block, Chebyschev,	Gaussian, Cubic
	Euclidean, Hamming, Mahalanobis,	Maximum Penalty: range [0.001, 1000]
	Minkowski, Spearman	
**Optimisation**	Bayesian	Bayesian
**Optimised Parameters**	K: 10	Kernel: Linear
	Distance Metric: Spearman	Max Penalty: 2.09
	Iterations: 30	Iterations: 30
**Cross-validation**	5-fold	5-fold

**Table 3:** REM Sleep Atonia Index (RAI) statistical parameters computed for healthy subjects (HS) and RBD. Subjects 5–7 (HS) are not included.

	HS	RBD
**Mean ± STD**	0.93 ± 0.14	0.44 ± 0.37
**Median**	0.945	0.40
**75th percentile**	1	0.8
**25th percentile**	0.3	0.04

**Table 4:** EMG spectral features (mean ± STD), computed for the healthy (HS) and RBD subjects (RBD) in the CAP Sleep Database. Mean Frequency (MF), Median Frequency (SEF50) and Spectral Edge Frequency at 95% (SEF95).

	HS	RBD
**MF (Hz)**	39.07 ± 13.62	47.53 ± 10.82
**SEF50 (Hz)**	34.73 ± 13.70	42.94 ± 12.24
**SEF95 (Hz)**	87.08 ± 23.90	101.42 ± 14.53

**Table 5:** 5-fold cross–validation performance of the two classifiers.

	Accuracy	Sensitivity	Specificity	PPV	FDR
K-NN	86.96%	93.33%	75%	87.50%	12.50%
SVM	82.61%	86.67%	75%	86.67%	13.33%

**Table 6:** Classification performance on the test set (30% of the total dataset).

	Accuracy	Sensitivity	Specificity	PPV	FDR	MAE
K-NN	81.82%	85.71%	75%	85.72%	14.28%	13.63%

**Table 7:** Detection performance of the two classification models, in terms of Mean Absolute Error (MAE) and Average Model Accuracy.

	MAE (%)	Model Accuracy (%)
**K–NN**	0	100%
**SVM**	5.55%	94.45%

**Table 8:** Euclidean Distance of RBD subjects in the CAP Database. Summary of the tested neighbourhood/reference combinations.

Neighbourhood/Reference	Euclidean Distance (Mean ± STD)	Max Distance	No. Subjects Outside Radius
**R${}_{1}$/S${}_{1}$**	5.35 ± 0.63	6.19	5
**R${}_{2}$/S${}_{1}$**	5.24 ± 0.59	5.80	7
**R${}_{2}$/S${}_{2}$**	5.02 ± 0.6	5.81	6

## Data Availability

The data presented in this study are available on request from the corresponding author. The data are not publicly available due to privacy or ethical reasons.

## Abbreviations

The following abbreviations are used in this manuscript:

ALN1	Average length of segments in Stage 1 Sleep
ALN2	Average length of segments in Stage 2 Sleep
ALREM	Average length of segments in REM Sleep
ALSWS	Average length of segments in Slow Wave Sleep
ARI	Arousal Index
CAP	Cyclic Alternating Pattern
CV	Cross-Validation
DI	Dissociation Index
ECG	Electrocardiogram
ECG	Electrocardiography
ED	Euclidean Distance
EEG	Electroencephalography
EMG	Electromyography
EOG	Electrooculography
FDR	False Discovery Rate
FNE	First Night Effect
HS	Healthy Subjects
HT	High Tier
IQR	Interquartile Range
iRBD	isolated REM Sleep Behaviour Disorder
KDE	Kernel Density Estimation
K-NN	K-Nearest Neighbour
LBD	Lewy Body Dementia
LT	Low Tier
MAE	Mean Absolute Error
MED	median
MF	Mean Frequency
ML	Machine Learning
MT	Moderate Tier
N1	Stage 1 Sleep
N2	Stage 2 Sleep
NFI	Non-REM Fragmentation Index
OSAS	Obstructive Sleep Apnea
PD	Parkinson’s Disease
PD-RBD	Parkinson’s Disease with REM Sleep Behaviour Disorder
PN1	Proportion of Stage 1 Sleep
PN2	Proportion of Stage 2 Sleep
PN3	Proportion of Slow Wave Sleep
PNR	Proportion of REM Sleep
PPV	Positive Predicted Value
PSD	Power Spectral Density
PSG	Polysomnography
RAI	REM Sleep Atonia Index
RBD	REM Sleep Behaviour Disorder
REM	Rapid Eye Movement
RFI	REM Fragmentation Index
RSWA	REM Sleep Without Atonia
SEF50	Median Frequency
SEF95	Spectral Edge Frequency at 95%
SOL	Sleep Onset Latency
STD	Standard Deviation
STI	Sleep Transition Index
SVM	Support Vector Machine
SWS	Slow Wave Sleep
TIB	Time in Bed
TST	Total Sleep Time
VHT	Very High Tier
WASO	Wake After Sleep Onset
WP	Wake Proportion
